# Role of Memory T Cells in Allograft Rejection and Tolerance

**DOI:** 10.3389/fimmu.2017.00170

**Published:** 2017-02-28

**Authors:** Gilles Benichou, Bruno Gonzalez, Jose Marino, Katayoun Ayasoufi, Anna Valujskikh

**Affiliations:** ^1^Center for Transplantation Sciences, Department of Surgery, Massachusetts General Hospital, Harvard Medical School, Boston, MA, USA; ^2^Department of Immunology, Lerner Research Institute, Cleveland Clinic, Cleveland, OH, USA

**Keywords:** memory T cells, allotransplantation, tolerance, heterologous immunity, transplant rejection, immune suppression, costimulation blockade

## Abstract

Memory T cells are characterized by their low activation threshold, robust effector functions, and resistance to conventional immunosuppression and costimulation blockade. Unlike their naïve counterparts, memory T cells reside in and recirculate through peripheral non-lymphoid tissues. Alloreactive memory T cells are subdivided into different categories based on their origins, phenotypes, and functions. Recipients whose immune systems have been directly exposed to allogeneic major histocompatibility complex (MHC) molecules display high affinity alloreactive memory T cells. In the absence of any prior exposure to allogeneic MHC molecules, endogenous alloreactive memory T cells are regularly generated through microbial infections (heterologous immunity). Regardless of their origin, alloreactive memory T cells represent an essential element of the allograft rejection process and a major barrier to tolerance induction in clinical transplantation. This article describes the different subsets of alloreactive memory T cells involved in transplant rejection and examine their generation, functional properties, and mechanisms of action. In addition, we discuss strategies developed to target deleterious allospecific memory T cells in experimental animal models and clinical settings.

## Introduction

Rapid and robust protective responses against previously encountered antigens are beneficial during infections, vaccinations, and tumor surveillance. Conversely, memory immune responses against donor antigens are detrimental in the context of transplantation and are commonly associated with poor graft outcome. The danger of preexisting donor-specific alloantibody (DSA) was recognized early in transplant history, and all transplant candidates are tested for the presence of serum DSA prior to transplantation. Despite well documented harmful effects of memory T cells in transplantation (1–4), the potential impact of such cells is mostly neglected while choosing treatment regimens. In this review, we initially outline characteristics of alloreactive memory T cells and their functions. We also describe existing and emerging strategies designed to delete or suppress memory T cells in transplant recipients. To conclude, we discuss future areas of investigation that may translate experimental knowledge of alloreactive memory T cells into clinical practice and thus improve transplant outcome in sensitized recipients.

## Basic Biology of Alloreactive Memory T Cells

### Origins of Alloreactive Memory T Cells

Laboratory rodents display low frequencies of memory T cells (5–10% of all T cells). In the absence of prior exposure to alloantigens, 1–10% of these memory T cells can react to allogeneic major histocompatibility complex (MHC) molecules *in vitro* (5). In mice, these cells called endogenous or natural alloreactive memory T cells recognize intact allogeneic MHC molecules through the direct allorecognition pathway (6, 7). It is likely that these memory cells are generated through the recognition of peptides from commensal bacteria or environmental antigens presented by self-MHC, which can mimic complexes formed by allogeneic MHC molecules bound to other peptides (8). Such antigen mimicry, named “heterologous immunity,” is well documented in both humans and experimental animal models. Humans and non-human primates raised in a non-sterile environment are exposed to more infectious and pro-inflammatory agents during their development and thereby likely to develop potent heterologous immunity (9). For instance, following an EBV infection, HLA-B8^+^ individuals can become sensitized to the allo-MHC molecule HLA-B4402 through antigen mimicry resulting from the presentation of some viral or parasitic peptides (10, 11).

In laboratory mice, direct sensitization with skin allografts or spleen cell immunization is a common approach for generating donor-reactive memory T cells. In humans, transplant patients can be sensitized from exposures to alloantigens such as previous transplants, pregnancies, and blood transfusions. Until now, only memory T cells recognizing intact alloantigens directly have been reported (2, 12). Yet, it is probable that sensitized patients exhibiting high titers of allospecific antibodies display memory T cells recognizing alloantigens indirectly as donor peptides–self-MHC complexes.

Memory T cells can also be generated through homeostatic proliferation in a lymphopenic environment, including potentially alloreactive and pathogenic T cells (13–15). Such homeostatically expanded memory T cells can impair tolerance induction to allografts (15–17).

The accumulation of alloreactive memory T cells may be influenced by the end stage organ disease or treatment common in transplant candidates. For example, prolonged exposure to dialysis increases the risk of developing alloreactive memory T cells (18). In addition, Sawinski et al. reported that low serum levels of 25-OH-vitamin D in dialysis patients correlates with the frequency of alloreactive memory T cells independent of age, gender, previous transplants, or time on dialysis (19).

### Location of Memory T Cells

**Figure 1:**
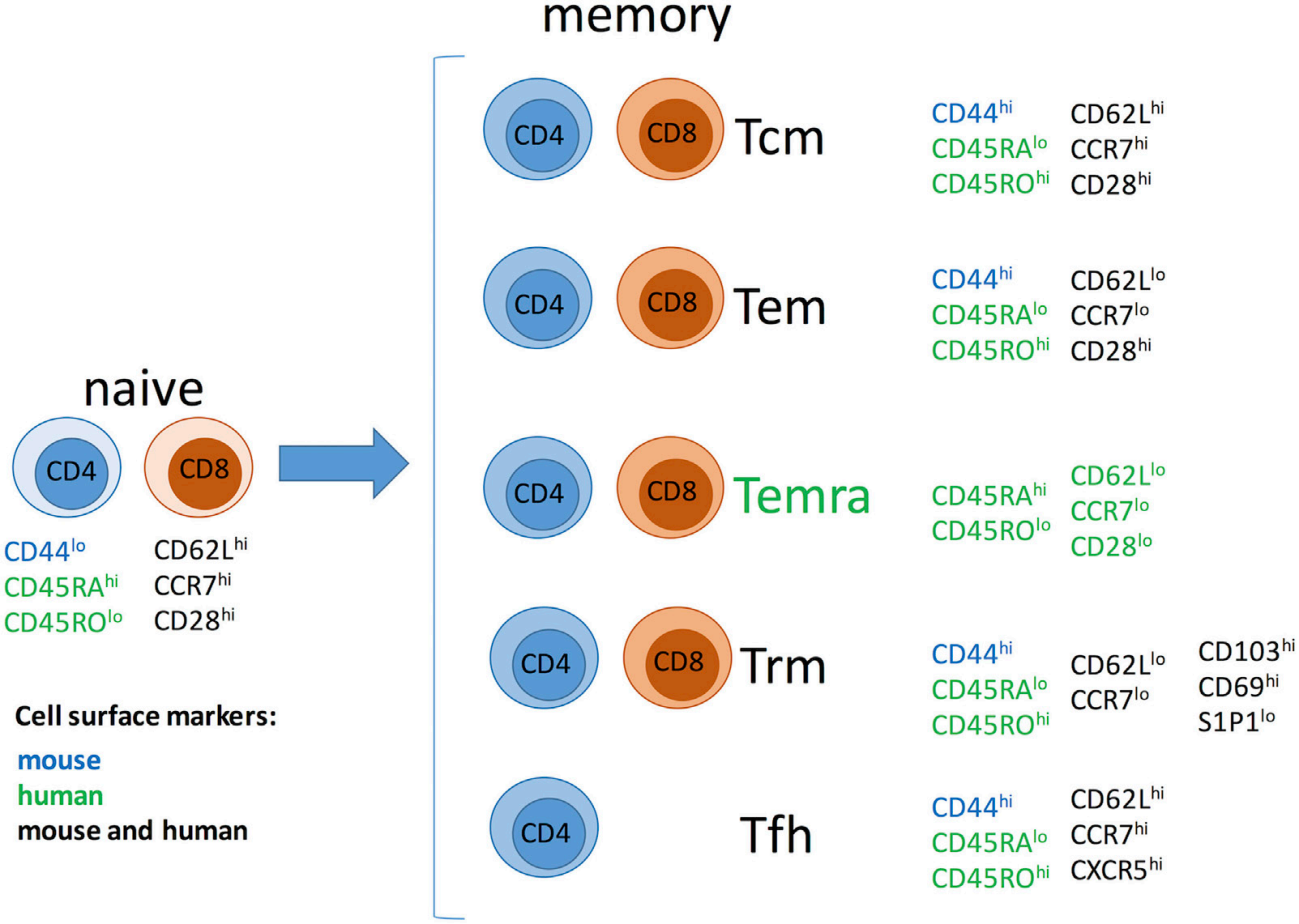
**Memory T cell subsets**. Abbreviations: Tcm, central memory T cells; Tem, effector memory T cells; Temra, terminally differentiated effector memory T cells; Trm, resident memory T cells; Tfh, follicular helper memory T cells.

Memory T cells have been traditionally divided into two major subsets with largely overlapping functions but distinct trafficking patterns (Figure 1). Central memory T cells (Tcm) express lymphoid homing markers CCR7 and CD62L, whereas effector memory T cells (Tem) are CCR7^−^CD62L^−^ but instead express molecules that promote migration into peripheral tissues (20–23). In humans, but not in mice, some memory T cells [terminally differentiated effector memory T cells (Temra)] re-express naive T cell surface marker CD45RA, while downregulating expression of CCR7, CD62L, and CD28, and represent a terminal stage of effector differentiation (21, 24, 25). Recent studies demonstrated that some T cells in peripheral tissues do not circulate and represent a distinct subset of tissue-resident memory T cells (Trm) (24, 26–28). Trm cells express early activation marker CD69 and αEβ7 integrin CD103 along with a number of tissue-specific chemokine receptors (26, 29–32). There is accumulating evidence that Trm cells play an important role in host protection against infections. It is conceivable that Trm cells of both donor and recipient origins may influence transplant outcome by facilitating GVHD or allograft rejection, respectively. However, the proportion of alloreactive T cells among Trm subset and the potential contribution of such cells following transplantation remain to be addressed. Another important type of memory T cells relevant to transplantation is CD4^+^CXCR5^hi^ follicular helper (Tfh) cells that reside in B cell follicles within secondary lymphoid organs and are essential for optimal B cell responses and antibody generation (33). As memory T cells in secondary lymphoid and non-lymphoid peripheral tissues are spared by antibody-mediated lymphoablation (34) Trm cells may be harder to control compared to circulating memory T cells.

### Low Activation Threshold and Resistance to Conventional Costimulatory Blockade

In the process of memory T cell differentiation, the T cell receptor and costimulatory signaling cascades are adjusted to ensure rapid activation of high magnitude upon antigen reencounter (35, 36). This results in the ability of memory T cells to respond to lower antigen doses with limited costimulation, i.e., to antigen presented by non-professional antigen-presenting cells (36–38). While this process is essential for host defense, it renders alloreactive memory T cells more dangerous in transplant settings. Numerous studies in animal models have demonstrated that donor-reactive memory T cells can induce allograft rejection despite interruption of essential costimulatory pathways, CD28/CD80/CD86 and CD40/CD154 (11, 15, 39–43).

## Contribution of Memory T Cells to Allograft Rejection and Tolerance

### Role in Allograft Rejection

During the past decade, studies investigating CD4^+^ versus CD8^+^ memory T cells revealed that these subsets contribute to allograft rejection through distinct mechanisms. Indeed, memory CD4^+^ T cells not only become effector cells upon reactivation, but also provide help for the robust activation of donor-reactive effector CD8^+^ T cells (40). These effector CD8^+^ T cells then are the main driving force behind allograft rejection facilitated by memory CD4^+^ T cells in heart-transplanted mice, and CD8^+^ T cell depletion or limiting their trafficking into the graft significantly extends allograft survival (40, 44).

While *de novo* responses by naïve T cells can be efficiently controlled by current immunosuppression, memory CD4^+^ T cells are resistant to these therapies and can provide help for the generation of DSA leading to alloantibody-mediated graft injury (40, 44). Recent studies in a mouse model of heart transplantation identified potential therapeutic targets to control CD40-independent DSA generation by memory CD4^+^ T cells. First, gamma interferon (IFNγ) secretion by memory helper T cells is required for *de novo* DSA generation (45). Second, CD40-independent helper functions of donor-reactive memory CD4^+^ T cells and heart allograft rejection were markedly inhibited by neutralizing B cell activating factor and a proliferation-inducing ligand, cytokines critical for B cell survival, activation, and differentiation (46).

The fate and functions of donor-reactive memory CD8^+^ T cells following transplantation are equally fascinating. Early direct contact of circulating memory CD8^+^ T cells with donor endothelium upregulates the expression of adhesion molecules and chemokines thus facilitating infiltration of recipient leukocytes into the graft (47, 48). A proportion of endogenous memory CD8^+^ T cells react to donor MHC class I molecules and can infiltrate cardiac allografts within hours after reperfusion. Once in the graft parenchyma, these memory CD8^+^ T cells proliferate extensively, upregulate the expression of ICOS, and secrete IFNγ in ICOS-dependent manner (49, 50). Although this early expression of effector functions was found to be insufficient to mediate allograft rejection (51), the potential danger of endogenous memory CD8^+^ T cells should not be underestimated. The approximation of clinical situation by increasing graft cold ischemia storage time enhanced effector functions of endogenous memory CD8^+^ T cells enabling them to promptly reject a cardiac allograft despite costimulatory blockade with CTLA4-Ig (52).

### Influence of Memory T Cells on Allograft Tolerance

In laboratory rodents, endogenous memory T cells generated through heterologous immunity have little ability to prevent tolerance induction given that hematopoietic chimerism and/or costimulation blockade regularly achieve tolerance of fully allogeneic transplants (53–55). In contrast, mice that have been sensitized to allogeneic MHC through transplantation or multiple viral infections become resistant to tolerance induction (11, 39, 56, 57). Moreover, naïve mice adoptively transferred with alloreactive memory T cells display similar resistance to tolerogenesis *via* hematopoietic chimerism or costimulation blockade (11, 39, 56, 57). Therefore, in laboratory rodents, antigen-induced rather than endogenous memory T cells prevent transplant tolerance. It is still unclear whether this difference relies on the low frequency of endogenous memory T cells or on the fact that these two subsets of memory T cells are different in nature.

The presence of memory T cells has been often correlated with poor outcomes in clinical transplantation. In humans, the presence of memory T cells pretransplantation has been associated with an increased risk for acute rejection of kidney transplants (2). However, while EBV- and CMV-specific memory T cells displaying alloreactivity have been detected in human transplant recipients, so far there is no indication that the presence of “heterologous immunity” in transplant recipients correlates with worse graft outcomes (10, 58–60).

Our laboratory showed that a sizable proportion of endogenous memory T cells found in peripheral blood, and secondary lymphoid organs of naïve cynomolgus monkeys are allospecific. Most Tem were CD8^+^CD95^+^CD28^−^ IFNγ-producing cells located in the spleen, peripheral blood, and bone marrow while IL-2-producing Tcm were primarily CD4^+^CD95^+^CD28^+^ and limited to the lymph nodes and spleen (12). Based upon this observation, we studied the influence of pretransplant memory T cell alloreactivity on rejection versus tolerance of kidney allografts in monkeys (61). A series of cynomolgus monkeys were conditioned [whole body and thymic irradiations + horse antithymocyte globulin (ATG) treatment] and received a combined kidney and bone marrow transplantation from the same allogeneic donor (62). The animals then received a short-term immunosuppression treatment comprised of anti-CD40L antibodies and cyclosporine A (62). This procedure resulted in a transient multilineage hematopoietic chimerism and achieved long-term survival of kidney allografts (>1 year) after withdrawal of immunosuppression in 70% of the monkeys (62). On the other hand, approximately 30% of the treated monkeys rejected their allograft in an acute fashion within 100–200 days posttransplantation (61). In this model, we observed that the vast majority of tolerant animals displayed low frequencies of donor-reactive memory T cells (61). It is noteworthy that no differences between homeostatic expansion of memory T cells were observed between monkeys which rejected or accepted kidney allografts (61).

Even though memory T cells are generally viewed as pathogenic in the context of transplantation, under certain circumstances, they demonstrate regulatory capacity and suppress deleterious pro-inflammatory immune responses. Krupnick et al. have reported that early infiltration of central memory CD8^+^ T cells is essential for lung allograft acceptance after treatment with CTLA4-Ig and anti-CD154 mAbs (63). Similarly, CD8^+^CD45RC^lo^ cells with regulatory properties have been described in rat models of solid organ transplantation and GVHD (64, 65). These findings raise a concern that lymphoablative approaches targeting memory T cells may interfere with allograft acceptance of certain types of transplants.

## Recent Developments in Targeting Alloreactive T Cell Memory

### Lymphoablation

Induction therapy is widely used in clinical transplantation to overcome the deleterious effects of preexisting donor-reactive immunity. Antibody-mediated lymphocyte depletion is most commonly used induction strategy, particularly in highly sensitized patients and in patients receiving marginal grafts (66–69). Although memory T cells are the primary targets of induction therapies, they are less susceptible to depletion than naïve T cells (70–73). T cells with an effector/memory phenotype are detectable after anti-CD52 mAb or ATG induction and are associated with acute rejection episodes in non-human primates and human transplant recipients (74, 75). In rodents, preexisting memory T cells rapidly recover following lymphocyte depletion with ATG and dominate anti-donor immune responses. The efficiency of memory CD4^+^ T cell depletion is generally lower than that of CD8^+^ T cells (34, 76–79). Additional depletion of residual CD4^+^ T cells severely impairs the recovery of memory CD8^+^ T cells after ATG treatment (80). Limiting CD4^+^ T helper signals during lymphoablation increases the efficacy of mATG in controlling memory T cell expansion and significantly extends heart allograft survival in sensitized recipients (80). These findings are consistent with previous observations describing a synergistic effect between ATG lymphoablation and costimulatory blockade (81, 82).

Alefacept, a fusion protein combining extracellular domain of LFA-3 with constant regions of human IgG1 (83–85). LFA-3 is a ligand for CD2, a molecule that is predominantly detected on human T and NK cells. As CD2 expression is upregulated on CD45RO^+^ effector/memory T cells, alefacept selectively depletes this subset and spares other T cell populations (86–88). Alefacept is currently being used in clinic for the treatment of severe psoriasis (89, 90) and is showing promise for targeting alloreactive effector/memory T cells in solid organ and bone marrow transplantation (91–95). Most importantly, pretransplant alefacept therapy synergizes with CTLA4-Ig presumably by targeting costimulatory blockade-resistant CD8^+^CD2^hi^CD28^−^ effector/memory T cells (91).

In addition to direct lymphoablation, manipulating T cell survival and homeostasis by regulating cell metabolic pathways may be a promising therapeutic strategy in transplantation. Recent studies suggest that immune cells subsets use different mechanisms of energy generation, and this information can be exploited to selectively target undesirable memory T cells [reviewed in Ref. (96)].

### Costimulatory Blockade

Belatacept, a second generation of CTLA4-Ig, is currently used in clinical transplantation to prevent allograft rejection and minimize the toxic side effects of calcineurin inhibitors (97). Despite reduced side effects and improved graft survival, belatacept-treated patients have higher rates of acute cellular rejection compared to CNI treatment (98, 99). As memory T cells are more resistant to the effects of CTLA4-Ig in animal transplantation models, it is possible that presensitized T cells could account for some belatacept-resistant rejection episodes. Indeed, terminally differentiated memory CD4^+^ and CD8^+^ T cells in humans (Temra) lose CD28 expression and become insensitive to the lack of CD28/B7 costimulation (100–104). Not surprisingly, increased numbers of both CD4^+^ and CD8^+^ CD28^−^ memory T cells are associated with a poor outcome in renal and lung transplant patients (105–108). A recent report by Espinosa et al. identified yet another population of CD57^+^CD4^+^ T cells as potential mediators of belatacept-resistant renal allograft rejection. These cells are more common in patients with kidney failure, express high levels of adhesion molecules CD2, LFA-1, and VLA-4, downregulate CD28, and produce IFNγ, tumor necrosis factor alpha (TNFα), and granzyme B consistent with effector/memory phenotype (109).

Recent reports suggest that the pedigree of alloreactive memory T cells in a given recipient may have important practical implications. Using three different pathogens to generate donor-reactive memory T cells in a mouse model of skin transplantation, Badell et al. demonstrated that the sensitivity of memory T cells to immunosuppression is dependent on their origin (110). In this study, Tcm with a less differentiated phenotype were most sensitive to the effects of costimulatory blockade. Consistent with these findings, *in vitro* comparison of CMV- and alloreactive T cells suggested that virus-specific fully differentiated T cells secreting IFNγ, TNFα, and IL-2 simultaneously are more resistant to the effects of CTLA4-Ig, whereas tacrolimus inhibits responses by both allo- and virus-specific T cells (111).

In addition to blocking CD28/B7 costimulation, CTLA4-Ig also prevents signaling through CTLA-4, which can have negative effects on generation and functions of regulatory T cells (Tregs) (112–117). To circumvent this problem, several antagonistic anti-CD28 mAbs and Ab F(ab′)2 fragments have been generated and showed promise in animal transplantation models (118–121). The selective effects of these reagents on memory T cell subsets and the potential pathogenicity of CD28^lo^ Temra cells during such therapies remain to be determined. Attempts to target another major costimulatory pathway, CD40/CD154, encountered early difficulties because of thromboembolic effects of anti-CD154 (CD40L) blocking antibodies (122). To avoid cross-linking CD154 that is highly expressed on platelets, an alternative approach has been the generation of non-activating anti-CD40 antibodies. Several such reagents have been successfully tested in non-human primate recipients of renal and islet allografts (123–128).

In addition to CD28/B7 and CD40/CD154 costimulation, several other costimulatory pathways may play a role in effector/memory T cell functions. Inhibition or genetic lack of ICOS/B7RP-1, CD134/CD134L, CD70/CD27, or CD137/CD137L improved allograft survival even in donor-sensitized recipients, or after delayed administration which allowed initial priming of donor-reactive T cells [reviewed in Ref. (129)]. It was revealed that these costimulatory pathways might control distinct aspects of the alloimmune response. For example, blocking anti-CD134L mAb inhibits proliferation of effector T cells while supporting the survival of Tregs (71, 130). Conversely, signaling through CD134 inhibits immunosuppressive properties of FoxP3^+^ Tregs and promotes allograft rejection (131, 132). ICOS/B7RP-1 blockade of resting memory CD4^+^ T cells inhibits their helper functions and decreases alloantibody production. In contrast, circulating memory CD8^+^ T cells are ICOS^lo^, but rapidly upregulate ICOS surface expression upon graft infiltration. These examples demonstrate that the complexity of costimulatory pathways governing alloimmune responses must be considered when costimulatory blockade is used as part of immunosuppression regimen.

### Limiting Trafficking of Alloreactive Memory T Cells

While preventing memory T cell entrance into graft tissue should improve transplant outcome, the attempts to neutralize chemokines or chemokine receptors such as CCR5 or CXCR3 did not live up to the initial expectations, most likely due to the redundancy of chemokine/receptor network. On other hand, reagents blocking LFA-1 (leukocyte function-associated antigen-1, an αLβ2 integrin) and VLA-4 (very late antigen-4, an α4β1 integrin) have been demonstrated to prolong allograft survival in experimental transplantation [reviewed in Ref. (133)]. Treatment with either anti-LFA-1 or anti-VLA-4 blocking mAbs prolonged skin allograft survival in a mouse model of costimulatory blockade-resistant rejection by memory CD8^+^ T cells (134). In another study, pretransplant treatment with anti-LFA-1 mAbs inhibited early infiltration of endogenous donor-reactive memory CD8^+^ T cells into cardiac allografts, and significantly prolonged allograft survival (135). These findings suggest that a short course of integrin blockade may be instrumental in controlling T cell memory while avoiding side effects of long-term treatments.

## Concluding Remarks

While other types of immunologic memory lymphocytes such as memory B cells, preexisting alloantibodies, and “innate memory” described for NK cells and macrophages can impact transplant outcomes, in this review, we focused exclusively on T cell memory. It is now firmly established that alloreactive memory T cells accelerate allograft rejection and prevent transplant tolerance. However, the implementation of accumulated experimental knowledge in clinical transplantation is impeded by several factors. First, the diagnostics of T cell allosensitization in transplant candidates is problematic. Due to heterogeneity in phenotype and functions of memory T cells, complementary tests will be required including analyses of cytokine producing, cytotoxic, and follicular helper T cells. The resulting information is likely to be complex and hard to use in clinical decision-making. Second, memory T cells in humans are sampled only in peripheral blood. So far, there is no information on pathogenicity of tissue-resident alloreactive memory T cells. Third, memory T cell susceptibility to immunosuppression may depend on their origins. As immunological histories of individuals are difficult to trace, the situation may arise when patients with similar T cell memory profile require distinct treatment strategies. Finally, despite rapidly accumulating data on alloreactive T cell memory, the discrepancies between animal models and transplantation in human patients are profound. Ideally, animal transplantation models approximating clinical situation should take into account frequencies of total and donor-reactive memory T cells in different species, time of graft cold ischemia storage, and the presence of DSA in recipient serum. Including these considerations into experimental design will facilitate the development of novel approaches to control memory T cells and improve transplant survival in sensitized recipients.

## Author Contributions

GB, AV, BG, JM, and KA wrote portions of the manuscript; GB and AV edited the manuscript and prepared it for submission.

## Conflict of Interest Statement

The authors declare that the research was conducted in the absence of any commercial or financial relationships that could be construed as a potential conflict of interest.

## Abbreviations

MHC, major histocompatibility complex; APC, antigen-presenting cell; Treg, regulatory T cell; IFNγ, gamma interferon; TNFα, tumor necrosis factor alpha; CTL, cytotoxic T lymphocyte; DC, dendritic cell; DSAs, donor-specific antibodies.
